# Cellular Injuries in *Cronobacter sakazakii* CIP 103183T and *Salmonella enterica* Exposed to Drying and Subsequent Heat Treatment in Milk Powder

**DOI:** 10.3389/fmicb.2018.00475

**Published:** 2018-03-13

**Authors:** Emilie Lang, Stéphane Guyot, Caroline Peltier, Pablo Alvarez-Martin, Jean-Marie Perrier-Cornet, Patrick Gervais

**Affiliations:** ^1^UMR PAM A 02.102 Procédés Alimentaires et Microbiologiques, Université de Bourgogne Franche-Comté/AgroSup Dijon, Dijon, France; ^2^Novolyze, Daix, France

**Keywords:** *Salmonella enterica*, *Cronobacter sakazakii*, membrane permeability, respiratory activity, heat treatment, drying

## Abstract

Because of the ability of foodborne pathogens to survive in low-moisture foods, their decontamination is an important issue in food protection. This study aimed to clarify some of the cellular mechanisms involved in inactivation of foodborne pathogens after drying and subsequent heating. Individual strains of *Salmonella* Typhimurium, *Salmonella* Senftenberg, and *Cronobacter sakazakii* were mixed into whole milk powder and dried to different water activity levels (0.25 and 0.58); the number of surviving cells was determined after drying and subsequent thermal treatments in closed vessels at 90 and 100°C, for 30 and 120 s. For each condition, the percentage of unculturable cells was estimated and, in parallel, membrane permeability and respiratory activity were estimated by flow cytometry using fluorescent probes. After drying, it was clearly observable that the percentage of unculturable cells was correlated with the percentage of permeabilized cells (responsible for 20–40% of the total inactivated bacteria after drying), and to a lesser degree with the percentage of cells presenting with loss of respiratory activity. In contrast, the percentages of unculturable cells observed after heat treatment were strongly correlated with the loss of respiratory activity and weakly with membrane permeability (for 70–80% of the total inactivated bacteria after heat treatment). We conclude that cell inactivation during drying is closely linked to membrane permeabilization and that heat treatment of dried cells affects principally their respiratory activity. These results legitimize the use of time–temperature scales and allow better understanding of the cellular mechanisms of bacterial death during drying and subsequent heat treatment. These results may also allow better optimization of the decontamination process to ensure food safety by targeting the most deleterious conditions for bacterial cells without denaturing the food product.

## Introduction

*Salmonella enterica* and *Cronobacter sakazakii* are Gram-negative, facultative anaerobic, motile, nonspore-forming bacteria that cause human disease (Beuchat et al., 2013). They are of major concern in the infant-food industry (Podolak et al., 2010), because ingestion of very few (10–100) *S. enterica* cells by young children causes severe illness (Rotger and Casadesús, 1999), and *C. sakazakii* can cause severe infections such as meningitis, bacteremia, and necrotizing enterocolitis in infants, with a death rate up to 80% and an infectious dose estimated 10^3^ cells (Friedemann, 2008; Strydom et al., 2012).

Foodborne pathogens encounter many stresses in food processing environments and food products (Wesche et al., 2009). Drying, which occurs during the production of low-moisture foods, is one of these stresses. It consists of a diminution of water activity (a_w_), which represents the available water for chemical and biochemical reactions (Iaconelli et al., 2015). During desiccation, water evaporation involves two main cellular stresses (Billi and Potts, 2002). The first corresponds to an increase in solute concentration and the osmotic pressure in the external environment, which induces a water release from the cell (Billi and Potts, 2002; Dupont et al., 2014). The second corresponds to complete water evaporation, where bacteria in a dried state undergo an oxidative stress because of exposure to atmospheric oxygen (Billi and Potts, 2002; Dupont et al., 2014). Bacteria have several active or passive mechanisms to resist osmotic stress, which enhance their survival and acclimation. The passive mechanisms, resulting from water outflow, involve molecular desiccation by refolding of molecules such as enzymes to protect them from active site alteration (Parsegian et al., 1995). They also involve a membrane phase transition from liquid to gel phase. The active mechanisms, which occur in the range of physiological a_w_ (between a_w_ of 0.999 and approximately 0.940, depending on the bacterial species), involve accumulation of ions and counterions inside the cell that reduce water loss before another response that permits the synthesis of compatible solutes, for example, trehalose for *S. enterica* (Rychlik and Barrow, 2005; Spector and Kenyon, 2012).

Bacteria in a dried state are more resistant to the decontamination processes, such as heat treatment, that are usually used (Laroche and Gervais, 2003; Fine et al., 2005). This resistance of bacteria in the dried state to heat is related to their very low cellular molecular mobility, which reduces the effects of further thermal treatments by preventing the disruption of disulfide and hydrogen bonds. This increased resistance may also be explained by active mechanisms that occur during the earlier stages of drying, which, as previously reported, also activate metabolic pathways that permit the modification of membrane composition and/or protein production, resulting in cross-protection (Rychlik and Barrow, 2005; Dancer et al., 2009; Shen and Fang, 2012; Spector and Kenyon, 2012). For example, a recent study reported that the *rpoS* gene, responsible for the general stress response, was involved in the cross-protection of desiccation-adapted *S*. Typhimurium against high temperature in aged broiled litter, showing the clear link between desiccation stress response and heat stress response (Chen and Jiang, 2017). In addition, drying increases the thermal resistance of several foodborne pathogens, including *S. enterica* or *C. sakazakii*, because of the production heat-shock proteins (HSP) during drying (Potts, 2001; Dancer et al., 2009; Li et al., 2012).

Heat is a classical and effective means to reduce microbiological load in food and is widely used in food industry (Lee et al., 1989). In liquid media, it has been well-managed for many years; processes such as pasteurization or sterilization are optimized and the mechanisms of bacterial inactivation in liquid media are well-understood. For Gram negative bacteria, heat treatment in liquid media is known to disrupt the outer membrane, resulting in irreversible damages (Chang et al., 2009). The inner membrane is also sensitive to thermal treatment, and heating causes perturbation of the biochemical functions of the membrane that are deleterious for bacterial cell (Russell, 2003; Guyot et al., 2010). Proteins are also denatured by heat treatment, which causes disruption of hydrogen and disulfide bonds and consequently the loss of protein function (Russell, 2003). These two mechanisms are involved in bacterial cells death during heat treatment in liquid media and result in a large scale inactivation of bacterial population. In contrast, the impact of heat treatment on dried foods is not well-known, and clarifying this will allow the optimization of heat treatment to preserve food qualities such as aroma, color, or vitamins, while assuring food safety. Public opinion easily accepts heat treatment, in contrast to acceptance of other types of treatment such as irradiation (Kume et al., 2009), and a better understanding of the mechanisms involved could lead to the establishment of optimal heating conditions by identification of the treatment parameters such as temperature that are most deleterious for bacterial cells.

In this study, we investigated the impact of drying and subsequent heat treatment on the membrane integrity and respiratory activity of *C. sakazakii, S. enterica* subsp. *enterica* serovar Typhimurium and serovar Senftenberg in milk powder. Propidium iodide (PI) and 5-cyano-2,3-ditolyl tetrazolium chloride were used in this study to assess the membrane integrity and the respiratory activity, respectively, permitting to visualize the impact of technological stresses on bacterial structure and functionality. Drying was performed to two different a_w_ levels (0.25 and 0.58, also used as control for heat treatment) and heat treatment was carried out at two temperatures (90 and 100°C) for 30 and 120 s. After treatment, the membrane integrity and respiratory activity of bacteria were investigated using flow cytometry with two fluorescent molecular probes.

## Materials and Methods

### Bacterial Cultures

*Salmonella enterica* subspecies *enterica* serovar Typhimurium DT104 DSM 10506, *Salmonella enterica* subspecies *enterica* serovar Senftenberg 775W DSM 10062, and *C. sakazakii* CIP 103183T strains were used for this study. *S. enterica* is especially involved in low-moisture foods outbreaks. More specifically, *S*. Typhimurium was chosen for its high thermal resistance in dried state and *S*. Senftenberg was chosen for its high thermal resistance. Also, *C. sakazakii* was chosen for its involvement in outbreaks with infant feeding, including infant low moisture foods. All cultures were stored in tryptic soy broth (TSB, Sigma-Aldrich, Saint-Quentin-Fallavier, France) with 20% glycerol (Sigma-Aldrich) at -80°C. For recovery, bacteria were inoculated on tryptic soy agar (TSA, Sigma-Aldrich) at 37°C for 24 h, after which five colonies of each bacterium were picked and placed in 50 mL of TSB and incubated for 8 h at 37°C. Suspensions were then diluted in 50 mL of new TSB to reach an optical density (OD) of 0.01 at 600 nm. Cultures in stationary phase were obtained after 20 h at 37°C.

### Inoculation of Milk Powder

The inoculation of the powder was based on a previous study to achieve a homogeneous inoculation (Lang et al., 2017). Briefly, cultures of 50 mL (OD_600_ = 2.14, 1.34, and 1.64 for *C. sakazakii, S*. Typhimurium, and *S*. Senftenberg, respectively) were centrifuged (3,400 *g*, 10 min at 25°C) and washed twice with 25 mL of phosphate buffered saline (PBS, Sigma-Aldrich). As a final step, the supernatant was removed and the cell pellets were weighed. Milk powder (26% fat, Régilait, Saint-Martin-Belle-Roche, France) was progressively added to the pellet at a 1:20 ratio (w_pellet_:w_powder_) and homogenized in a mortar to obtain an inoculated milk powder with an a_w_ close to 0.8, measured using an a_w_-meter (Aqualab, Dardilly, France).

### Drying Process

To dry the inoculated milk powder, we used hermetically sealed boxes with saturated salt solutions at the bottom that regulated the a_w_ and as a consequence the atmosphere relative humidity (RH). Potassium acetate to achieve an a_w_ of 0.25 (corresponding to a RH of 25%) and sodium bromide (both from Sigma-Aldrich) to achieve an a_w_ of 0.58 (corresponding to a RH of 58%) were used. When boxes were closed, convection of atmospheres was maintained using a ventilator (Sunon, Radiospare, France) as described previously (Lemetais et al., 2012). Approximately, 2 g of milk powder inoculated with each bacterial strain was spread on each of four small Petri dishes, which were placed without their lids inside hermetic boxes for 16 h to reach the final a_w_ level. All drying experiments were performed at room temperature.

### Thermal Treatment

Samples of dried inoculated milk powder (0.1 g) were placed into 0.2 mL tubes and treated at two different holding temperatures (90 and 100°C) for 30 and 120 s and then cooled to 4°C to arrest the impact of thermal treatment using a thermocycler (Bioer, France). The come-up and cooling times correspond to 7 and 15 s, respectively. Milk powder was rehydrated by addition of 1 mL of PBS and agitation for 30 s.

### Bacterial Suspension Preparation for Analyses After Drying and Heating

All rehydrated bacterial suspensions were washed after treatment and before analysis for culturability and cytometry analyses to remove the milk. Briefly, one milliliter of a 10^-2^ dilution (10^6^ bacteria/mL) was washed in an equal volume of PBS by centrifugation (3,200 *g*, 5 min, 25°C) and serial dilutions were performed from this washed suspension. The culturability of bacteria was estimated by the spread-plating method after incubation in TSA media for 24 h at 37°C and recorded as colony-forming units/mL. As culturability and flow cytometry results were compared, they were both compared in percentage. Loss of culturability after drying was expressed as a percentage of the initial bacterial cells, i.e., as (N_1_/N_0_) × 100, where N_1_ represents the dried bacterial cell concentration and N_0_ represents the initial bacterial cell concentration (bacteria contained in a pellet reported to the weight of added milk powder). The logarithmic loss of culturability after drying was also expressed as log_10_(N_1_/N_0_). The loss of culturability after heat treatment was expressed as a percentage of the initial dried bacterial cells, i.e., as (N_2_/N_1_) × 100, where N_2_ represents the final bacterial cell concentration and N_1_ represents the dried bacterial cell concentration at the corresponding a_w_ level. The loss of culturability after heating was also expressed as log_10_(N_2_/N_1_). The detection limit of this method was a 5 log decrease. The washed 10^-2^ dilution (10^6^ bacteria/mL) of each sample was used for cytometric analyses.

### Flow Cytometry Analysis

#### Labeling of Bacterial Cells

Propidium iodide (Invitrogen, France) and 5-cyano-2,3-ditolyl tetrazolium chloride (CTC, from the BacLight RedoxSensor kit, Invitrogen, France) were used in this study to assess the membrane integrity and the respiratory activity, respectively.

Propidium iodide is a fluorescent intercalating agent of DNA that penetrates permeabilized membranes, allowing evaluation of the membrane permeability of cells. CTC is a fluorescent marker for the detection of bacterial respiration through the activity of dehydrogenase, an enzyme of the respiratory chain. In the cellular electron transport system, tetrazolium salts function as artificial redox partners in place of oxygen, which is the final electron acceptor; thus, CTC indicates the respiration activity by production of a fluorescent formazan product that is impermeable to intact cellular membranes (Rodriguez et al., 1992; Créach et al., 2003; Hatzinger et al., 2003; Berridge et al., 2005).

Propidium iodide stock solutions were prepared for use at 1 mg/mL and CTC stock solution was made up at 50 mM in filtered distilled water. Then, 300 μL of bacterial suspension was labeled independently with 3 μL of PI and 3 μL of CTC stock solutions (final probe concentrations of 10 μg/mL PI and 5 mM CTC), at room temperature for 20 min for PI and at 37°C for 30 min for CTC.

#### Flow Cytometry Conditions

Each labeled bacterial suspension was analyzed directly after incubation using an Accuri C6 cytometer (BD Accuri, France) with excitation at 488 nm. A detection threshold was set on forward scatter signals to reduce electronic and small-particle noise. The optical filter used for both dyes was a 585 nm bandpass filter. Typically, signals from 10,000 cells were acquired and analyzed for each sample using the BD Accuri CSampler software (v 1.0.264.21). The percentages of permeabilized and non-respiring bacteria were adjusted in relation to the control obtained in TSB and washed as described previously for the samples (i.e., 100% of permeabilized bacteria for PI and 100% of non-respiring bacteria for CTC). Assuming that permeabilized and non-respiring bacteria are definitively inactivated, we expressed the corresponding results as a percentage of inactivated bacteria only. As a consequence, the number of inactivated bacteria was expressed as 100% and percentages of permeabilized and non-respiring bacteria were adjusted accordingly. The gating strategy used is presented in **Supplementary Figure S1**.

### Statistical Analyses

All experiments were performed independently in triplicate. The effects of each variable of the drying and the thermal treatment were evaluated for each bacterium by analysis of variance (ANOVA) using R software (R Development Core Team, 2008). Significance was evaluated when the *p*-value ≤ 0.05; in this case a Tukey’s HSD (Honest Significant Difference) test was performed to reveal significant differences among the conditions. To demonstrate the eventual correlation between the loss of culturability and physiological injuries (membrane permeability and respiratory activity), a Pearson correlation test was performed.

## Results

The results are presented in two parts: first, the effect of drying and, second, the effects of heating of dried bacteria on the culturability, membrane integrity, and respiratory activity of *C. sakazakii, S*. Typhimurium, and, *S*. Senftenberg.

### Impact of Drying on Bacterial Cells

**FIGURE 1:**
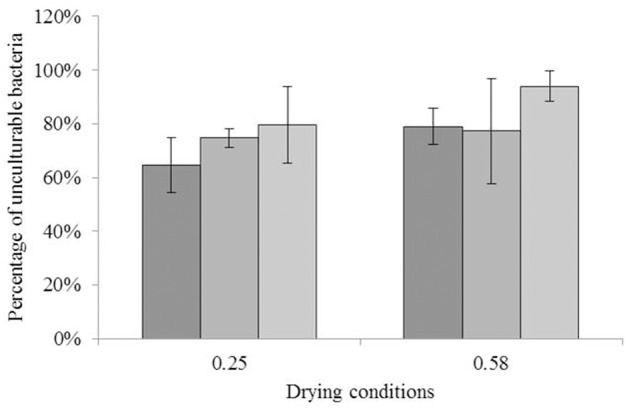
Impact of drying on culturability. Impact of drying (16 h) on culturability of each species; the bars from darker to lighter show the percentages of unculturable *Cronobacter sakazakii, Salmonella* Typhimurium, and *Salmonella* Senftenberg cells as a function of the water activity level of drying. The equivalent logarithmic inactivation is indicated above each bar. Error bars represent the standard deviation obtained using independent triplicates. The corresponding logarithmic inactivation numbers are presented in **Supplementary Table S1**.

The impact of drying to different a_w_ on the culturability of each strain is illustrated in **Figure 1**. ANOVA did not reveal any significant differences between the values. For *C. sakazakii*, 64.5% (-0.45 log) and 78.9% (-0.68 log) of the initial number of bacteria were unculturable after drying to a_w_ = 0.25 and a_w_ = 0.58, respectively. For *S*. Typhimurium, the unculturable bacteria at a_w_ = 0.25 and a_w_ = 0.58 represented 74.7% (-0.60 log) and 77.2% (-0.65 log), respectively, of the initial number of bacteria. For *S*. Senftenberg, 79.5% (-0.69 log) and 93.0% (-1.22 log) of the initial number of bacteria were unculturable after drying to a_w_ = 0.25 and a_w_ = 0.58, respectively.

**FIGURE 2:**
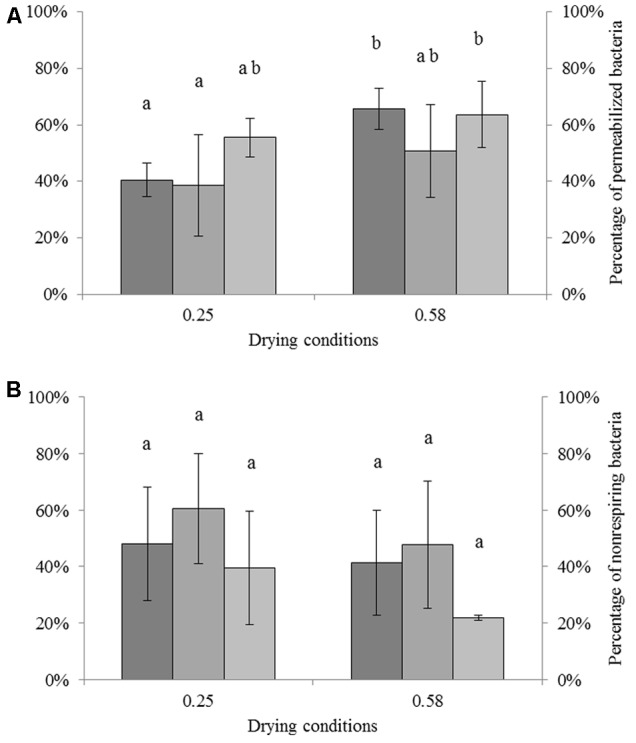
Impact of drying on permeability and respiratory activity of *Cronobacter sakazakii, Salmonella* Typhimurium, and *Salmonella* Senftenberg cells. The number of unculturable cells was adjusted to 100% (presented on the left). **(A)** Impact of drying (16 h) on membrane permeability of each species reported as percentage of unculturable cells. From darker to lighter, bars represent the percentages of permeabilized *C. sakazakii, S.* Typhimurium, and *S.* Senftenberg cells (right axis). **(B)** Impact of drying (16 h) on respiratory activity of each species reported as percentage of unculturable cells. From darker to lighter, bars represent the percentages of non-respiring *C. sakazakii, S.* Typhimurium, and *S.* Senftenberg cells (right axis). Error bars represent the standard deviation obtained using independent triplicates.

The impact of drying on the membrane permeability of each strain at different a_w_ is shown in **Figure 2A**. Results were calculated by adjusting the percentage of unculturable cells (presented in **Figure 1**) to 100%. For *C. sakazakii, S*. Typhimurium, and *S*. Senftenberg dried to a_w_ = 0.25, the percentages of permeabilized cells were 40.6, 38.7, and 55.6% of the unculturable cells, respectively. After drying to a_w_ = 0.58, the percentages of permeabilized cells were 65.8, 50.7, and 63.6% of the total unculturable cells for *C. sakazakii, S*. Typhimurium, and *S*. Senftenberg, respectively. ANOVA showed a significant effect of the a_w_ on the percentage of permeabilized cells. Small differences were detected for all strains between drying to a_w_ = 0.25 and a_w_ = 0.58, namely 18.2, 12.4, and 8.0% for *C. sakazakii, S*. Typhimurium, and *S*. Senftenberg, respectively. The percentages of permeabilized cells were slightly higher after drying to a_w_ = 0.58 than to a_w_ = 0.25.

The impact of drying on respiratory activity, which is related to the loss of capacity to respire, of each strain at different a_w_ levels is presented in **Figure 2B**. These results were also calculated by normalizing the unculturable cells to 100%. For *C. sakazakii, S*. Typhimurium, and *S*. Senftenberg dried to a_w_ = 0.25, the percentages of non-respiring cells were 48.1, 60.7, and 39.7% of unculturable cells, respectively. After drying to a_w_ = 0.58, the percentages of non-respiring cells were 41.3, 47.6, and 21.8% of unculturable cells for *C. sakazakii, S*. Typhimurium, and *S*. Senftenberg, respectively. ANOVA showed no significant differences between conditions, although small differences of 6.8, 12.8, and 17.9% for *C. sakazakii, S*. Typhimurium, and *S*. Senftenberg, respectively, were detected between those dried to a_w_ = 0.58 and to a_w_ = 0.25.

**FIGURE 3:**
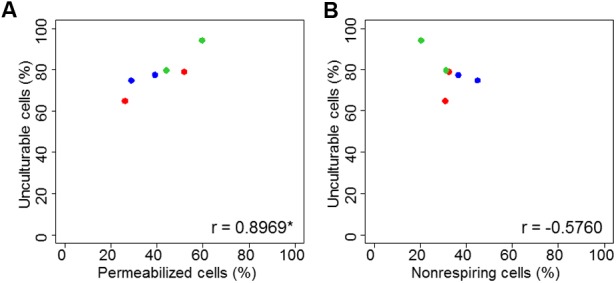
Correlation plot of unculturable bacterial cells during drying as function of membrane permeability **(A)** and respiratory activity **(B)**. Correlation plots were proposed for *C. sakazakii* (red), *S.* Typhimurium (blue), and *S.* Senftenberg (green). “c” is the correlation coefficient and the “^∗^” indicates if correlation is significant, both obtained with Pearson correlation test.

A comparison of the percentages of permeabilized and non-respiring bacteria after drying is particularly interesting. For drying to a_w_ = 0.25, both alterations were equally represented. At a_w_ = 0.58, the proportions of unculturable cells were slightly higher in permeabilized bacteria than in non-respiring bacteria. ANOVA revealed a significant effect of a_w_ but no differences between the two dyes or between bacterial strains. To complete ANOVA, a Pearson correlation test was performed. The link between permeabilized or non-respiring cells and unculturable cells is presented in **Figure 3**. The unculturable bacteria was well-linked with permeabilized and non-respiring cells. The correlation was significant (Pearson correlation test, *p* < 0.05) between unculturable and permeabilized bacteria.

### Impact of Heat Treatment on Bacterial Cells

**FIGURE 4:**
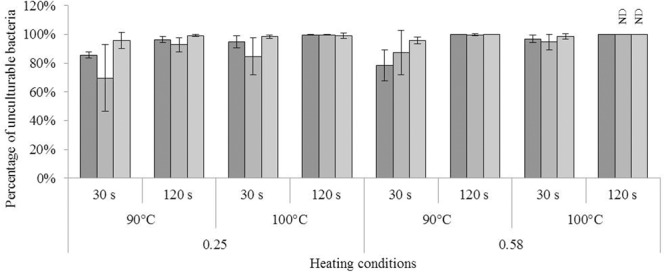
Impact of heating in dried state on culturability of *Cronobacter sakazakii, Salmonella* Typhimurium, and *Salmonella* Senftenberg cells. Impact of heat treatment on culturability of each species; from darker to lighter, bars represent the percentages of unculturable *C. sakazakii, S.* Typhimurium, and *S.* Senftenberg cells as a function of the heating conditions, namely the water activity of the milk powder (0.25 or 0.58), heating temperature (90 or 100°C), and time of treatment (30 or 120 s). The equivalent logarithmic inactivation is indicated above each bar. Error bars represent the standard deviation obtained using independent triplicates. The corresponding logarithmic inactivation numbers are presented in **Supplementary Table S1**.

**FIGURE 5:**
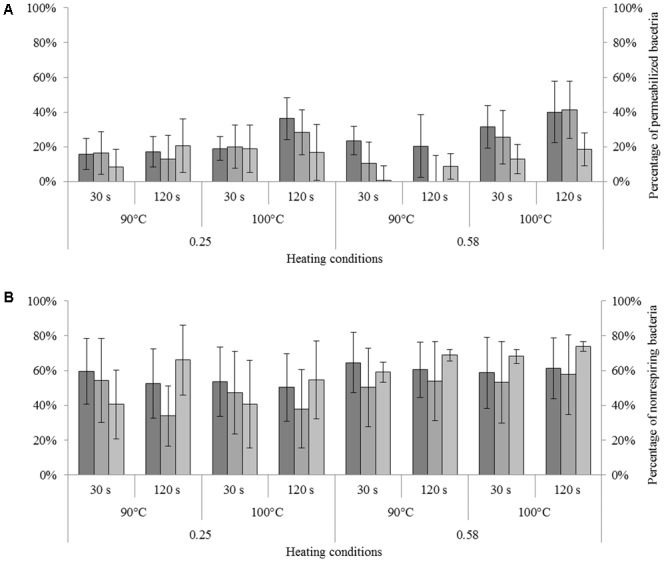
Impact of heating in dried state on permeability and respiratory activity of *Cronobacter sakazakii, Salmonella* Typhimurium, and *Salmonella* Senftenberg cells. The number of unculturable cells was adjusted to 100% (presented on the left). **(A)** Impact of heating on membrane permeability of each species reported as a percentage of unculturable cells. From darker to lighter, the bars represent the percentages of permeabilized *C. sakazakii, S.* Typhimurium, and *S.* Senftenberg cells (right axis). **(B)** Impact of heating on respiratory activity of each species reported as a percentage of unculturable cells. From darker to lighter, bars represent the percentages of non-respiring *C. sakazakii, S.* Typhimurium, and *S.* Senftenberg cells (right axis). Error bars represent the standard deviation obtained using independent triplicates.

The impact of heat treatment on bacterial culturability, membrane permeability, and respiratory activity is shown in **Figures 4, 5**. The results were obtained after treatment in closed vessels at 90 or 100°C for 30 or 120 s. The results obtained after heat treatment were adjusted for the results obtained after drying to discern only the impact of heating.

The impact of heat treatment on bacterial culturability is presented in **Figure 4**. For each bacterium, the proportion of unculturable bacteria, i.e., the loss of culturability, was greater at a_w_ = 0.58. Similarly, unculturability was greater after treatment at the higher temperature (100°C) and for the longer time (120 s). For each bacterium, the lowest percentage of unculturable bacteria was observed in milk powder at a_w_ = 0.25 treated at 90°C for 30 s (see **Supplementary Table S1** for results of logarithmic inactivation). After treatment of milk powder at a_w_ = 0.58 for 120 s at 100°C, culturability of both *Salmonella* serovars was below the detection limit of the method (**Figure 4**). ANOVA demonstrated significant effects of temperature, time, and strain on bacterial inactivation.

The impact of heat treatment on the membrane permeability of each strain under different heating conditions is illustrated in **Figure 5A**. The results were calculated by adjusting the proportion of unculturable cells to 100% and subtracting the effect of drying; i.e., the percentage of permeabilized cells observed after drying was subtracted from the percentage of permeabilized cells observed after drying and heat treatment to identify only the impact of heat treatment on dried bacteria. ANOVA revealed a significant effect of strain and time of treatment on the percentage of permeabilized cells; *S*. Senftenberg contained the fewest permeable bacteria after heat treatment. Membrane permeability increased with increasing treatment time. For all strains, the percentage of permeabilized cells within the unculturable cells was less than 20% except after heat treatment at 100°C for 120 s, when an increase in membrane permeability was observable. The proportion of permeabilized cells was greater after heat treatment of a milk powder at a_w_ = 0.58 than at a_w_ = 0.25 under the same conditions. For example, for *S*. Typhimurium treated at 100°C for 120 s, the percentages of permeabilized bacteria were 28.5 and 41.3% for milk powders at a_w_ = 0.25 and a_w_ = 0.58, respectively.

The impact of different heat treatment conditions on the respiratory activity of each strain is presented in **Figure 5B**. Results were calculated by adjusting the unculturable cell number to 100% and subtracting the effect of drying. More non-respiring bacteria were detected after heat treatment at a_w_ = 0.58 than at a_w_ = 0.25. For all strains, the percentages of non-respiring bacteria within the unculturable cells were between 40 and 75%.

We also compared the percentages of permeabilized and non-respiring bacteria, and observed that the percentages of non-respiring bacteria were significantly (2- to 5-fold; *p* < 0.05, ANOVA) higher than the percentages of permeabilized bacteria. For example, for *C. sakazakii* heated at 90°C for 30 s in a milk powder at a_w_ = 0.58, the unculturable bacteria included 23.5 and 64.5% permeabilized and non-respiring bacteria, respectively.

**FIGURE 6:**
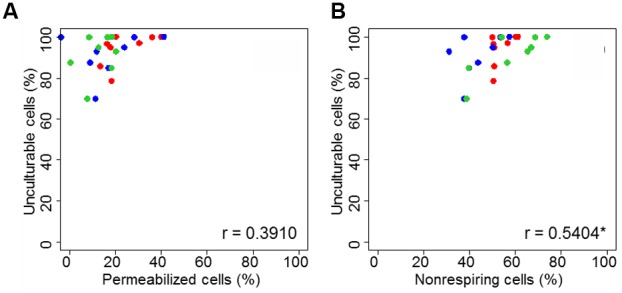
Correlation plot of unculturable bacterial cells during heat treatment in dried state as function of membrane permeability **(A)** and respiratory activity **(B)**. Correlation global was proposed for *C. sakazakii* (red), *S.* Typhimurium (blue), and *S*. Senftenberg (green). “c” is the correlation coefficient and the “^∗^” indicates if correlation is significant (*p* < 0.05), both obtained with Pearson correlation test.

The link between permeabilized or non-respiring cells and unculturable cells is presented in **Figure 6**. The correlation was significant (Pearson correlation test, *p* < 0.05) between unculturable and non-respiring bacteria.

## Discussion

In this study, the loss of culturability of three foodborne pathogens was studied after drying and then after subsequent heat treatment. The percentages of bacterial cells with permeabilized membranes and loss of respiratory activity after treatment were also evaluated to understand better the origin of the loss of culturability.

In our study, we used a higher inoculation load than natural contamination of milk powder, commonly comprised between 1 and 6 CFU/g. It was already known that microbial load impact the bacterial population response to stress, for which a high inoculation lead to a higher inoculation ratio than with a lower inoculation level (Burnett et al., 2000; Lin and Beuchat, 2007). Nevertheless, if we used such low inoculation level, the detection of cells would be more difficult. Consequently, a high contamination level was used in our study to ensure observation of bacterial inactivation via CFU measurement and facilitate flow cytometry analyses.

Since many years, flow cytometric analyses in bacteriology increased. By using different probes, it also showed its usefulness to study the impact of environmental perturbation on a bacterial cell (Winson and Davey, 2000; Wang et al., 2010). Nevertheless, this analysis method necessitates sheath liquid. In our study, we worked in dried food and consequently a rehydration step is obligatory to observe the impact of environmental perturbation on bacterial cells by culturability and flow cytometry analyses. The rehydration is known to impact the culturability of the microorganisms but also the passage of dried to hydrated state could interfere with different cellular structures. This obligatory step could perturb our observation as well as any observation performed with other methods.

The loss of culturability of each strain after drying did not differ after drying to a_w_ = 0.25 and a_w_ = 0.58 (**Figure 1**). Nevertheless, the loss of culturability was greater after heat treatment at a_w_ = 0.58 than at a_w_ = 0.25 (**Figure 4**), as shown in a previous study. It is already well-known that a decrease in a_w_ increases the thermal resistance of microorganisms, including *S. enterica, Lactobacillus plantarum*, and *Saccharomyces cerevisae* (Laroche et al., 2005). For example, for heat treatment at 72°C in sucrose solution, the time necessary to obtain three-log decrease in *S.* Typhimurium dropped from 4.0 min at a_w_ = 0.65 to 1.0 min at a_w_ = 0.90. In our study, *C. sakazakii* appeared to be the most resistant species, and was still detectable after a heat treatment at 100°C for 120 s at a_w_ = 0.58, under which conditions neither *Salmonella* serovar was detectable (**Figure 4**). Indeed, consistent with this, *C. sakazakii* is often considered to be the most resistant *Enterobacteriaceae* in dried state (Arroyo et al., 2009). In addition, the *S*. Senftenberg 775W strain in this study had already been demonstrated as the most resistant strain of *S. enterica*. Indeed, its great resistance is well-known in liquid media since 1969 (Ng et al., 1969). It was confirmed in other type of product as aged chicken litter and beef/turkey blended patties (Murphy et al., 2002; Chen et al., 2013). Our results regarding the relative resistance of this strain are in accordance with these previous studies.

The use of PI for the measurement of bacterial membrane permeability is well-established (Stiefel et al., 2015). Contrary to the situation for eukaryotic cells, there is no evidence that PI is incorporated by non-permeabilized bacterial cells (Davey and Hexley, 2011). For CTC labeling, dead bacteria are non-fluorescent because of direct enzyme denaturation or because of the escape of formazan across the permeabilized membrane. As a consequence, it is possible to be certain that PI-labeled bacteria are truly permeabilized while, in contrast, CTC-nonlabeled bacteria can represent three types of cells: (i) non-respiring, (ii) permeabilized but respiring, and (iii) non-respiring and permeabilized. Consequently, the presence of permeabilized but respiring bacteria within the CTC-nonlabeled population could lead to an overestimation of the number of non-respiring bacteria.

After drying, no significant difference was detected between the percentage of permeabilized and of non-respiring bacteria (**Figure 2**). Consequently, it is possible that both types of injury occur equally during drying. However, it is also possible that the CTC-nonlabeled cells are in fact permeabilized cells from which formazan have been released. The Pearson correlation test (**Figure 3**) brings complementary information to ANOVA as it permits to observe the evolution of one parameter versus another, but correlation is not sufficient alone to conclude regarding the quantitative occurrence of one phenomenon (permeabilized or non-respiring bacteria) for both treatments (drying and heating). No significant correlation was observed between unculturable and non-respiring bacteria, meaning that the interference suspected between CTC and membrane permeabilization is not consistent for all treatments, especially for a moderate drying at a_w_ = 0.58, which could be due to the fact that for this condition dye could stay in intracellular media. The link between cell inactivation during drying and membrane permeabilization was studied for *Saccharomyces cerevisiae* by Simonin et al. (2007), who observed that membrane permeabilization largely occurs after an osmotic stress in water-glycerol solution and subsequent rehydration (Simonin et al., 2007). Similar results have been reported for *E. coli*, in which the formation of vesicles was observed after dehydration (Mille et al., 2003). The authors of that study proposed that intense dehydration resulted in membrane invagination followed by vesicle formation, which can then lead to membrane permeabilization and disruption after rehydration. Their proposed hypothesis for the cellular lysis or permeabilization during rehydration was the relative lack of membrane surface area because of vesicle formation. As a consequence, drying could effectively promote bacterial membrane permeability (Mille et al., 2003; Zoz et al., 2016).

The unexplained percentage of unculturable bacteria in our study could be attributed to the presence of viable but non-culturable cells (VBNC) (Oliver, 2005; Fakruddin et al., 2013; Ramamurthy et al., 2014). Notably, *S. enterica* is known to produce VBNC during drying, because of activation of the pathway for VBNC production, which involves the RpoS transcription factor that is also essential for the osmotic stress response (Ramamurthy et al., 2014). Bacteria can resuscitate from the VBNC state in the presence of an ideal ratio of carbon source and inorganic elements (Ramamurthy et al., 2014). This unexplained component of inactivated bacteria could also be to the result of other injuries not analyzed here such as damage to DNA, which is known to be oxidized during drying (Potts, 1994).

The results of our study clearly showed that the loss of culturability after heat treatment is better explained by a loss of respiratory activity than by a loss of membrane integrity (**Figure 5**). In addition to ANOVA results, correlation is significant between unculturable and non-respiring bacteria (**Figure 6**). Although several causes of loss of culturability of microorganisms after heating in liquid media have been identified, to our knowledge, no results for pathogen inactivation after heat treatment in dried state have been reported. Some studies were performed at low water activity in liquid media where cell inactivation was associated to an increase in membrane permeabilization (Beuchat and Scouten, 2002; Mille et al., 2002; Aljarallah and Adams, 2007; He et al., 2011). Guyot et al. (2010) demonstrated the importance of structure, fluidity, and the maintenance of membrane integrity during heat treatment to preserve the cell culturability of *E. coli* in liquid media (Guyot et al., 2010). Chang et al. (2009) presented an electron micrograph analysis showing that heat-shock treatment caused damage and disruption to *C. sakazakii* cells, notably in the outer membrane (Chang et al., 2009). In our study, enzyme denaturation was clearly related to cell inactivation after heat treatment. Indeed, depending on the duration and severity of the heat stress, the accumulation of defective proteins can result in the bacteria cell inactivation. Importantly, it was shown previously that in liquid mainly heating promotes protein denaturation and/or aggregation (Russell, 2003). Hence, alterations of biomolecules can explain most bacterial inactivation during heat treatment in a dried state.

The unexplained percentage of unculturable cells, between 30 and 60% (difference between the percentage of inactivated and non-respiring bacteria), could be attributed to other alterations occurring in bacterial cells. For example, it could be to the result of the alteration of some vital biomolecules that are more sensitive to heat than the two molecular models studied here. It is well-known that ribosomal denaturation can be a cause of bacterial inactivation during heating and can prevent the *de novo* protein synthesis during bacterial recovery (Rosenthal and Iandolo, 1970; Tolker-Nielsen and Molin, 1996). Finally, this effect could also be the result of VBNC production during the drying step.

## Conclusion

This study analyzed and compared the damage to cellular components that led to death during drying and subsequent heat treatment of three foodborne pathogens in milk powder at two different a_w_ and two heating temperatures. The results showed that membrane permeability (means between 40 and 70% approximately) is clearly implicated in bacterial inactivation during drying, while respiratory activity is probably less affected. In contrast, the results clearly showed that loss of respiratory activity (means between 40 and 70% approximately) is mainly responsible for bacterial inactivation during heat treatment in a dried state. Overall, these results allowed us to demonstrate that these two types of process commonly used in the food industry involve different principal causes of cell death. Notably, the major cause of death appears to differ during heat treatment in dried and liquid states (i.e., membrane permeabilization or enzyme/protein denaturation, respectively). An understanding of these phenomena and the role of a_w_ and temperature should permit the optimization of food processing to ensure food safety by identifying the most deleterious conditions for bacterial cells during both types of treatment. Indeed, because bacterial inactivation after heat treatment is related to protein denaturation, a time–temperature scale seems to be well-adapted to enhance bacterial inactivation. Nevertheless, because bacterial inactivation after drying is related to bacterial membrane permeabilization, a rapid variation in a_w_ level could preferentially enhance bacterial inactivation. However, the results of this study concern only three foodborne pathogens dried and heated in milk powder, and the conclusions must be confirmed by further studies on other species and substrates.

## Author Contributions

EL, SG, CP, PA-M, J-MP-C, and PG: the acquisition of the data for the work or the analysis and interpretation of data for the work. Drafting the work or revising it critically for important intellectual content. Final approval of the version to be published. Agreement to be accountable for all aspects of the work in ensuring that questions related to the accuracy or integrity of any part of the work are appropriately investigated and resolved.

## Conflict of Interest Statement

The authors declare that the research was conducted in the absence of any commercial or financial relationships that could be construed as a potential conflict of interest.

## Supplementary Material

FIGURE S1 Gating strategy for flow cytometry analysis. (^∗^) In the case of PI, the labeled cells were permeabilized cells. In the case of CTC, the labeled cells were the respiring cells.

TABLE S1 Logarithmic inactivation corresponding to unculturable percentage for each bacterium and each treatment condition.
