# Supplementary figures and images for: Cellular Injuries in Cronobacter sakazakii CIP 103183T and Salmonella enterica Exposed to Drying and Subsequent Heat Treatment in Milk Powder
Source: Front Microbiol. 2018 Mar 13;9:475. doi: 10.3389/fmicb.2018.00475 (PMC5859370; doi:10.3389/fmicb.2018.00475)

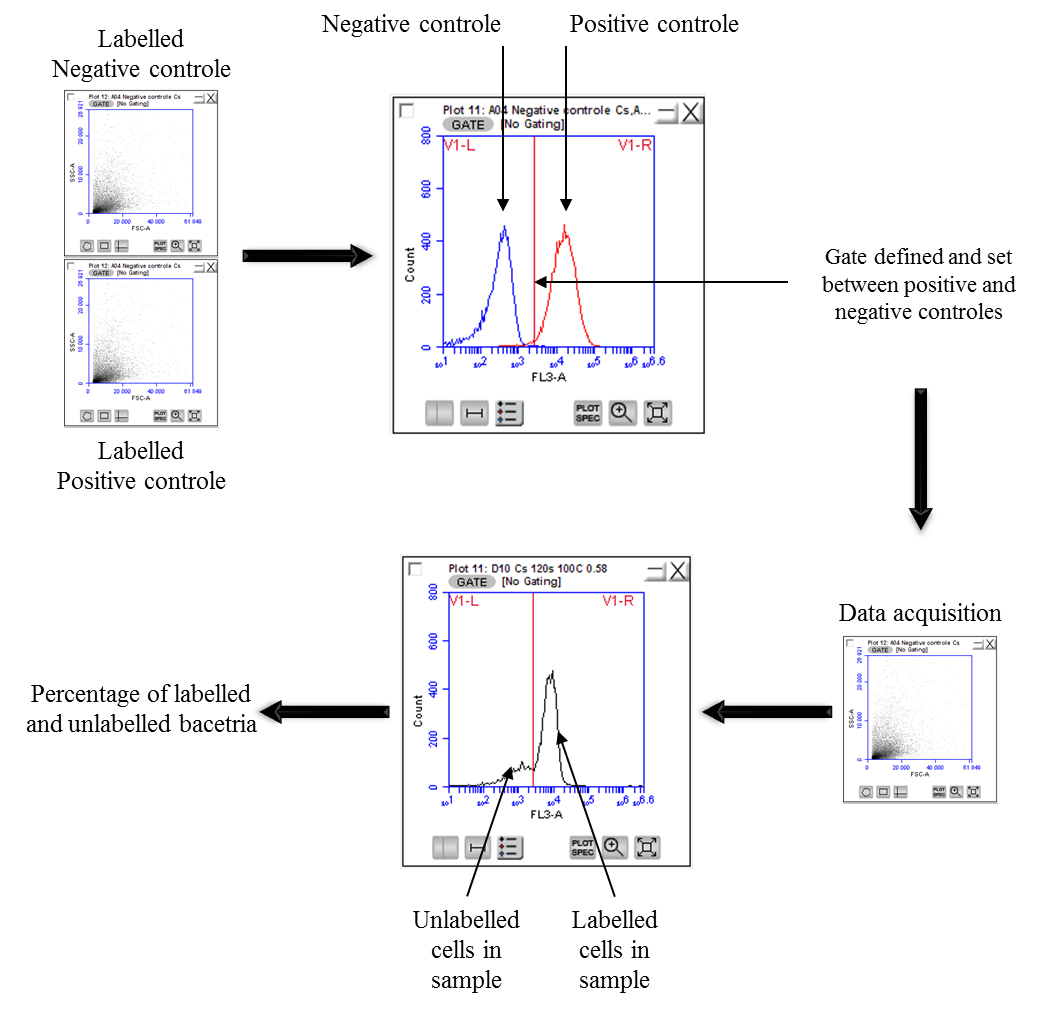

Supplement: FIGURE S1 — Gating strategy for flow cytometry analysis. (∗) In the case of PI, the labeled cells were permeabilized cells. In the case of CTC, the labeled cells were the respiring cells. [file Image_1.tif]
